# Supplementary material for: Patterns of Intron Gain and Loss in Fungi
Source: PLoS Biol. 2004 Nov 30;2(12):e422. doi: 10.1371/journal.pbio.0020422 (PMC532390; doi:10.1371/journal.pbio.0020422)
Supplement: Table S1 — Also available at http://genes.mit.edu/NielsenEtAl/. (4.3 MB ZIP). [file pbio.0020422.st001.zip › NielsenEtAl/html/370.html]

AN5872.1.NCU05295.1.MG07130.1.FG06159.1


```
 CLUSTAL W (1.82) Multiple Sequence Alignments - Introns Inserted


Sequence 1: NCU05295.1	245 aa
Sequence 2: MG07130.1	245 aa
Sequence 3: FG06159.1	243 aa
Sequence 4: AN5872.1	246 aa
Alignment Length: 246 aa
Number Identitical Residues: 205 aa
Alignment Score (without introns) 7983


MG07130.1 	MFMARSEYD1RGIN2TFSPEGRLFQVEYSLEAIKLGSTAIG~VATSEGVVLGVEKRVTST
NCU05295.1	MFMARSEYD1RGIN2TFSPEGRLFQVEYSLEAIKLGSTAIG~VATSEGVILGVEKRVTST
FG06159.1 	--MANPASD~RGIN~TFSPEGRLFQVEYSLEAIKLGSTAIG~VATSEGVILGVEKRVTST
AN5872.1  	MFIARSEYD1RGIN2TFSPEGRLFQVEYSLEAIKLGSTAIG0VATSEGVILGVEKRVTST
          	  :*..  * **** ************************** *******:**********

MG07130.1 	LLETSSVEKIVEIDRHIGCAMSGLQADARSMVEHARVECQSHAFNYNEDLRVESCTQAIC
NCU05295.1	LLETSSVEKIVEIDRHIGTAMSGLQADARSMIEHARVECQSHAFNYNEKLRVESCTQAIC
FG06159.1 	LLETSSVEKIVEIDRHIGCAMSGLQADARSMVEHARVESQSHAFNYNEPLRVESCTQAIC
AN5872.1  	LLEASSVEKIVEIDQHIGCAMSGLQADARSLVEHARVETQNHAFHYAEPLRVESCTQAIC
          	***:**********:*** ***********::****** *.***:* * ***********

MG07130.1 	DLALRFGESADGEESIMSRPFGVALLIAGYDEDGPQL~YHAEPSGTFYRYDAKAIGSGSE
NCU05295.1	DLALRFGESADGEESIMSRPFGVALLIAGYDEDGPSL~YHAEPSGTFYRYDAKAIGSGSE
FG06159.1 	DLALRFGEGADGEETIMSRPFGVALLIAGFDEDGPQL~FHAEPSGTFYRYDAKAIGSGSE
AN5872.1  	DLALRFGETGDDEESVMSRPFGVALLIAGIDEDGPQL2YHAEPSGTFYRYDAKAIGSGSE
          	******** .*.**::************* *****.* :*********************

MG07130.1 	GAQAELQNEYHK0SLSITDAETLVLKTLKQVMEEKLDAKNVQLASVTKEKGFRIYTDEEM
NCU05295.1	GAQAELQNEYHK0SLTLEDAETLVLKTLKQVMEEKLDAKNVQLASVTKDKGFRIYTDEEM
FG06159.1 	GAQAELQNEYHK0SLTLIDAETLVLKTLKQVMEEKLDAKNVQLASVTKEKGFRIYTDEEM
AN5872.1  	GAQAELQNEYHR~SLTLAEAETLVLKTLKQVMEEKLDAKNVQLASVTKEKGFRIYNDEEM
          	***********: **:: :*****************************:******.****

MG07130.1 	ATVVSRLPAN-
NCU05295.1	AAVVARLPAN-
FG06159.1 	AAVVERLPAN-
AN5872.1  	GRAVAQLGGNQ
          	. .* :* .*.
```
